# The protective role of competitive sports in reducing suicidality amongst youth athletes

**DOI:** 10.3389/fpsyg.2025.1591178

**Published:** 2025-06-04

**Authors:** Paola R. Sparagana, V. Claire Clark, Whitney Herge, Emily J. Stapleton

**Affiliations:** ^1^Department of Psychology, Scottish Rite for Children, Frisco, TX, United States; ^2^Department of Psychiatry, University of Texas Southwestern, Dallas, TX, United States

**Keywords:** adolescent mental health, competitive sports, suicidality, suicide screening, youth athletes

## Abstract

**Introduction:**

Suicidality is a critical concern among young people, and a prevalent concern in athletes. This study aimed to investigate the rate of suicidality among youth athletes in sports medicine clinics and to evaluate the risk for suicidality in this population.

**Methods:**

A retrospective chart review of 8,599 patients (10–18 years old) seen between 2018 and 2022 was conducted using the Ask Suicide-Screening Questionnaire (ASQ). Analyses examined relationships between ASQ responses and demographic, clinical, and sport-related variables.

**Results:**

Amongst athletes, higher competition levels were associated with lower rates of suicidality than those competing at lower levels (*p* = 0.0162). While female sex was associated with increased suicidality overall, this was not significant within the athlete subgroup.

**Discussion:**

Higher levels of sport competition were associated with lower rates of suicidal ideation, suggesting a potential protective effect.

## 1 Introduction

Suicide is the second leading cause of death among American children aged 10 to 14 years (Health, 2024; Prevention, 2022) and the prevalence of mental health disorders and suicidality (i.e., risk of suicide, indicated by suicidal ideation, intent, and/or history of suicidal behavior) are increasing in our youth (Health, 2024). According to death certificate data, the suicide rate among youth aged 10 to 24 years rose from 6.8 to 10.7 per 100,000 between the years 2000 and 2018 (Curtin, 2020). Moreover, there has been a 31% increase in mental health-related emergency department visits since 2019 among American adolescents (Yard et al., 2021).

Early identification and treatment of individuals at risk for suicide is a primary suicide prevention strategy (Group, 2018), and understanding risk factors for youth suicide is a critical component of risk identification. Factors contributing to suicide risk are multifactorial and include individual risk factors (e.g., previous suicide attempt(s), current or historical experience of mental health symptoms, current or historical experience of abuse, substance misuse, physical illness, chronic pain, personality factors, and experience of stressful life events), relationship factors (e.g., experience of bullying, social isolation, family or peer conflict, and family history of suicide), and environmental and social factors (e.g., barriers to accessing health care, access to lethal means, stigma associated with seeking mental health care, and systemic trauma or marginalized experiences; Prevention, 2024a). Female sex has also been identified as a risk factor for mental health symptoms and suicidality (Prevention, 2024b; Roh et al., 2024). Supportive relationships with caregivers and feeling a sense of connection to family, friends, and one’s community are known protective factors against youth suicide (Prevention, 2024a).

Research in sports populations has demonstrated mixed evidence regarding the impact of sport participation on mental health. On the positive side, certain studies have identified that youth who play sports demonstrate lower rates of depression and anxiety symptoms, stress, and psychological difficulties (Eime et al., 2013; Hoffmann et al., 2022; Logan et al., 2019; Murray et al., 2021; Panza et al., 2020; Sabo et al., 2005), as well as report higher levels of emotional identification and regulation skills, higher quality of life, more positive social relationships and social skills, and increased self-esteem compared to non-sport participating youth (Eime et al., 2013; Fernandes et al., 2024; Hoffmann et al., 2022; Logan et al., 2019; Vella, 2019). Regarding suicidality specifically, several studies have identified benefits to physical activity and sports engagement for suicidal risk. One longitudinal study found that middle and high school sports participants demonstrated a decreased likelihood of suicidal ideation compared to non-athlete peers (Taliaferro et al., 2011). Sabo et al. found that high school athletic participation was associated with decreased likelihood of suicidal ideation, and reduced odds of planning a suicide attempt in females (Sabo et al., 2005). Similarly, Roh et al. found that higher participation frequency in physical activity was associated with more positive mental health outcomes, whereby the rate of suicidal behavior decreased as the frequency of over 60 min of physical activity and strength training increased (Roh et al., 2024).

Alternatively, studies have also demonstrated mental health risks associated with sport participation. Specifically, sport participation has been linked to increased stress, lower self-esteem, increased negative mood states, and disordered eating behaviors, as well as substance abuse, burnout, and exposure to maltreatment and abuse (DiFiori et al., 2014; He et al., 2018; Logan et al., 2019). Certain environmental and personal characteristics appear to moderate the impact of these risk factors, including coaching climate, sports culture (e.g., expectations for body type, hazing), parental expectations and influence, training volumes, scheduling demands, and personality types (Logan et al., 2019).

In addition to the environmental and personal characteristics impacting mental health, sport-specific variables (e.g., team versus individual sport and specialization level) also significantly influence the variability of mental health outcomes in youth athletes. Participation in youth team sports is associated with fewer anxiety and depressive symptoms, and improved overall psychosocial health in adulthood, compared to both non-sport and individual sport participants (Fernandes et al., 2024; Hoffmann et al., 2022; Kunitoki et al., 2023). Findings on the benefits of participation in individual sports are mixed. Whereas Fernandes et al. found higher levels of emotional functioning and regulation in youth individual sport participants compared to non-sport participants, the overall mental health and well-being benefits were below team sport participants (Fernandes et al., 2024). Hoffman et al. did not find that individual sport participation was protective for mental health and psychosocial functioning (Hoffmann et al., 2022). Moreover, Hoffmann et al. (2022) found that athletes participating exclusively in individual sports tend to exhibit an increased prevalence of mental health problems when compared to team sport and non-sport youth, specifically with increased anxiety and depression, withdrawal, social problems, and attentional problems.

Sport specialization and participation in sports at higher levels have become increasingly popular in youth sports and have also demonstrated an increased risk for poorer psychosocial functioning and mental health. Specifically, Watson et al. found that female youth athletes who specialized in a single sport reported lower ratings of quality of life and sleep, both of which are often associated with poorer mental health and well-being (Watson et al., 2021). Specialized athletes are also at greater risk for injury, burn-out, social isolation, and mental health concerns due to the high physical, psychological, and social demands of sport specialization (Brenner et al., 2019; Weber et al., 2018; Wylleman et al., 2004).

Given the variability evident in the existing literature regarding the role of sport participation on youth mental health, this study aims to expand our understanding of sport-specific protective and risk factors associated with youth mental health concerns, especially related to suicidality. Bolstering our understanding of the mental health challenges within various youth sports populations given their unique experiences (e.g., individual versus team, specialization status, level of competition), will allow for more effective early identification and intervention. To our knowledge, the prevalence of suicidality between sport participation involvement and types has not been studied in youth. Therefore, the purpose of this study is to expand the understanding of suicidality in pediatric sports medicine and identify youth sport populations at increased risk for suicide. Given current literature on sport participation and mental health, it was hypothesized that females would demonstrate an overall higher risk prevalence for suicidality, in both sport and non-sport participating youth, and that individuals participating in youth sports would demonstrate an overall lower risk prevalence for suicidality relative to youth who do not participate in sports. Within youth athletes, we hypothesized that athletes participating in team sports would demonstrate a lower prevalence of suicidality compared to those in individual sports. Lastly, we hypothesized that highly specialized athletes would demonstrate an increased prevalence of suicidality compared to athletes at lower levels of specialization.

## 2 Materials and methods

This study was approved by the local Institutional Review Board (IRB; #STU-2020-1374). Informed consent was not required and was waived by the IRB as this study was a retrospective chart review and did not require additional procedures or interactions with patients.

### 2.1 Participants

A consecutive review of patients who had been formally diagnosed with a pediatric orthopedic and/or sports medicine condition and were seen in the institution’s sports medicine clinics between September 1, 2018 and August 4, 2022 was conducted. Participants were included if they were between the ages of 10–18 years and had completed an Ask Suicide-Screening Questionnaire (ASQ). Patients who did not complete the ASQ were excluded from the study. Completion of all other questionnaires, forms, or assessments used in this study did not affect the patient’s inclusion in the study.

### 2.2 Procedure

Patient data was obtained by conducting a diagnosis-based query with the hospital’s Health Information Management and the Billing Department for patients treated within the hospital’s sports medicine clinics who completed the ASQ. Data collected from the electronic medical record (EMR) included demographic, clinical, and sport-related variables, as well as patient responses to the self-report ASQ. Upon presentation to the sports medicine clinic, patients were comprehensively assessed through physical examination and sport-specific assessments, as well as questionnaires assessing their overall mental health (including anxiety, and suicidality), pain, daily functioning, and socioeconomic factors. All patients who present to the sports medicine facility over the age of 10 are administered a suicide screener every 6 months consist with Joint Commission on Accreditation of Healthcare Organizations’ National Patient Safety Goal for suicide prevention in health care settings. If patients endorse positive suicidality on any of the screener questions, they are evaluated by a licensed clinical social worker for a brief suicide safety assessment and given recommendations for further care, if indicated.

Demographic variables collected included: age at presentation, sex, race, ethnicity, insurance status, and school grade at presentation. Clinical variables collected included: visit provider (non-operative, operative, sports neurologist), visit type, reason for visit (body part and type of complaint), date of injury, and primary sports medicine diagnosis at conclusion of the visit. Sport-related variables were collected, which included sport participation (athlete or non-athlete), competition level (recreational, school, club/select, travel, or other), athlete type (single- or multi-sport athlete), sport participation type (individual, team, or both individual and team sport), primary sport, and additional sports played.

### 2.3 Measures

The Ask Suicide-Screening Questionnaire (ASQ; Horowitz et al., 2012) is a validated suicide risk screening tool for medical patients ages 8 years and older. It consists of four “Yes” or “No” questions that are used to screen for current and historical suicidal ideation and behavior. The four questions assess current thoughts of being better off dead, current wishes to die, current suicidal ideation, and any past suicide attempts. If the patient answers “Yes” to any of the four questions, or refuses to answer, then they are considered a positive screen for suicidality. If the patient answers “Yes” to any of the first four questions, a fifth question is asked to assess the immediacy of suicide risk.

### 2.4 Statistical analysis

Statistical analysis was conducted using R software (version 4.1.3, R Core Team, 2021) and SPSS (version 29). Descriptive statistics including frequencies, means, and standard deviations were calculated across all variables. The Shapiro–Wilk test was conducted to assess the normality of the variables. Depending on the results, either T-tests or Mann–Whitney U tests were used to evaluate statistically significant differences in continuous variables, including age and days since injury, between groups (e.g., athletes vs. non-athletes and ASQ positive vs. ASQ negative). The Chi-square or Fisher’s Exact test was used to compare categorical variables with ASQ responses amongst the entire population, athletes, and non-athletes. Categorical variables included sex, race, ethnicity, school level, athlete type (single/multi-sport), competition level, and chronicity of injury (acute/chronic). Logistic regression was used to identify predictors of a positive ASQ response, examining factors such as sex, anxiety scores, and competition level to determine their likelihood of association with suicidal ideation amongst the general population, athletes, and non-athletes. Backward selection was used to refine the logistic regression to include only the significant predictors. Statistical significance was concluded when *p* < 0.05.

## 3 Results

### 3.1 Descriptive statistics

A total of 8,599 patients between the ages of 10 and 18 (M = 12 ± 2.13) years were included for analysis. Patients were reflective of the study region in sex, race, and ethnicity (Table 1). A total of 5,714 patients (66.4%) identified as athletes, with the remainder identifying as non-athletes.

**Table 1:** Patient demographics and sport characteristics.

Variable	*N* (%)^*^
Race	
American Indian or Alaska Native	33 (0.38%)
Asian	431 (5.01%)
Black or African American	1,055 (12.27%)
Native Hawaiian or Other Pacific Islander	8 (0.09%)
White	6,361 (73.97%)
Other	268 (3.12%)
Multiracial	425 (4.94%)
Ethnicity	
Hispanic or Latino	2,080 (24.19%)
Non-Hispanic or Latino	6,469 (75.23%)
Sex assigned at birth	
Female	4,475 (52.04%)
Male	4,124 (47.96%)
Insurance plan	
Commercial insurance	5,843 (73.91%)
Government insurance	1911 (24.17)
Uninsured/no insurance/self-pay	152 (1.92)
Athlete status	
Athlete	5,712 (66.44%)
Non-athlete	724 (8.42%)
Competition level	
Recreational	578 (12.48%)
School	1,692 (36.52%)
Club/Select/Travel	2,194 (47.36%)
Other	3 (0.06%)
Sport participation type	
Individual sport	1,225 (21.46%)
Team sport	3,041 (53.27%)
Both individual and team sport	1,443 (25.28%)
Athlete type	
Multi-Sport Athlete	2,591 (45.38%)
Single-Sport Athlete	3,118 (54.62%)
Primary sport type^**^	
Basketball	710 (12.44%)
Baseball/Softball/T-Ball	739 (12.94%)
Football	791 (13.86%)
Soccer	1,102 (19.30%)
Volleyball	404 (7.08%)
School level	
Elementary	673 (9.85%)
Middle School	2,626 (38.44%)
High School	3,474 (50.85%)
College	57 (0.83%)
Not in School	2 (0.03%)

* Patients were not required to respond to every question.

** “Primary Sport Type” categories consist of the top 5 most reported sports.

Patients primarily sought medical treatment for knee-related issues (42.54%), followed by ankle issues (11.08%). Of the total population, 54.23% reported an identifiable injury as the reason for seeking medical treatment, 42.92% reported pain, and only 1.67% reported both pain and injury as the reason for seeking medical treatment. The most frequently reported physician-assigned medical diagnoses were knee related, with 2,294 patients (26.7%) diagnosed with a type of knee injury and 855 patients (9.94%) diagnosed with knee pain. Most patients (67.8%) reported having an acute problem lasting 90 days or less (Table 2).

**Table 2:** Patient clinical variables.

Variable	*N* (%)^*^
Visit type^**^	
Acute visit	4,428 (51.49)
New patient visit	2033 (23.64)
Follow-up visit	1,636 (19.03)
Concussion new patient visit	386 (4.49)
Visit provider	
Non-operative sports medicine physician	5,439 (63.25)
Sports medicine surgeon	3,081 (35.83)
Sports medicine neurologist	79 (0.92)
Reason for visit—body part^***^	
Knee	3,658 (42.54)
Ankle	953 (11.08)
Shoulder/Collarbone	633 (7.36)
Hip	502 (5.84)
Head	449 (5.22)
Reason for visit—complaint	
Injury	4,663 (54.23)
Pain	3,691 (42.92)
Both injury and pain	144 (1.67)
Other	101 (1.17)
Primary diagnosis^***^	
Knee injury	2,294 (26.68)
Knee pain	855 (9.94)
Ankle injury	672 (7.81)
Status post knee surgery	422 (4.91)
Back/spine pain	374 (4.35)
Chronicity^****^	
Acute	3,531 (67.8)
Chronic	1,678 (32.2)

* Patients were not required to respond to every question.

** “Visit Type” categories consist of the top 4 most reported visit types.

*** “Reason for Visit—Body Part” and “Primary Diagnosis” categories consist of the top 5 most reported body parts and diagnoses, respectively.

**** >90 days since injury is considered to be “chronic”.

### 3.2 Athletic participation

When evaluating patients who identified as athletes, soccer was the most frequently reported primary sport (19.3%). Nearly half of all athletes (47.4%) reported they compete at a club, select, or travel level (Table 1).

When comparing athletes to non-athletes, significant differences between the groups were identified (Table 3). Differences were identified between athletes and non-athletes in age (13.8 ± 2.07 vs. 14.2 ± 2.28 years, *p* < 0.0001), sex (51.5% male vs. 61.1% male, *p* < 0.0001), ethnicity (77.3% Non-Hispanic vs. 65.5% Non-Hispanic, *p* < 0.0001), and insurance provider (77.4% commercial insurance vs. 52.3% commercial insurance, *p* < 0.0001). Athletes were more likely to be seen by a non-operative sports provider (75.5% vs. 65.8%; *p* < 0.0001). No other differences were identified between the athlete and non-athlete subgroups.

**Table 3:** Comparison of demographics and clinical variables in athletes vs. non-athletes.

Variable	Athletes *N* (%)	Non-Athletes *N* (%)	*p*-value
Sex assigned at birth			
Female	2,942 (51.49%)	442 (61.05%)	< 0.0001^*^
Male	2,772 (48.51%)	282 (38.95%)	
Race			
American Indian or Alaska Native	26 (0.46%)	2 (0.28%)	N/R^**^
Asian	293 (5.13%)	54 (7.46%)	
Black or African American	686 (12.01%)	77 (10.64%)	
Native Hawaiian or Other Pacific Islander	7 (0.12%)	0 (0.00%)	
White	4,231 (74.07%)	529 (73.07%)	
Other	182 (3.19%)	32 (4.42%)	
Multiracial	287 (5.02%)	30 (4.14%)	
Ethnicity			
Hispanic	1,279 (22.40%)	247 (34.12%)	<0.0001^*^
Non-Hispanic	4,411 (77.26%)	474 (65.47%)	
Decline to answer	19 (0.33%)	3 (0.41%)	
Insurance plan			
Commercial insurance	4,087 (77.42%)	351 (52.31%)	<0.0001^*^
Government insurance	1,132 (21.44%)	299 (44.56%)	
Uninsured/no insurance/self-pay	60 (1.14%)	21 (3.13%)	
Visit provider			
Non-operative sports medicine physician	4,316 (75.53%)	476 (65.75%)	<0.0001^*^
Sports medicine surgeon	1,377 (24.10%)	244 (33.70%)	
Sports medicine neurologist	21 (0.37%)	4 (0.55%)	
Reason for visit—body part^***^			
Knee	2,120 (37.1)	426 (58.8)	N/R^**^
Ankle	397 (6.95)	17 (2.35)	
Shoulder/Collarbone	473 (8.28)	56 (7.73)	
Hip	394 (6.90)	21 (2.90)	
Foot	397 (6.95)	17 (2.35)	
Reason for visit—complaint			
Injury	2,755 (48.2)	355 (49.0)	0.6880
Pain	2,820 (49.4)	349 (48.2)	
Both injury and pain	110 (1.93)	14 (1.93)	
Primary diagnosis			
Knee injury	1,443 (25.3)	287 (39.64)	N/R^**^
Knee pain	669 (11.7)	125 (17.27)	
Ankle injury	564 (9.87)	57 (7.87)	
Back/spine pain	328 (5.74)	20 (2.76)	
Shoulder injury	272 (4.76)	34 (4.70)	

* Indicates significance at α = 0.05.

** N/R = no p-value was recorded for this variable.

*** “Reason for Visit—Body Part” categories consist of the top 5 most reported body parts.

### 3.3 Suicidal ideation prevalence

Of the 8,599 patients who completed the ASQ, 117 (1.36%) patients screened positive for suicide risk. No significant differences were found in race or ethnicity distribution between ASQ-positive and ASQ-negative groups. Regarding insurance status, there was a significant difference between ASQ-positive and ASQ-negative groups (*p* = 0.0262), with a higher percentage of ASQ-positive patients (35.19%) covered by government insurance compared to ASQ-negative patients (24.02%).

No significant differences were found in primary diagnosis, reason for visit—body part, or complaint type between ASQ-positive and ASQ-negative groups. Significant differences in ASQ response were observed across visit providers. Among ASQ-positive patients, the majority were seen by non-operative providers (69.23%), followed by operative providers (26.50%) and sports neurology providers (4.27%; *p* = 0.0016).

### 3.4 Sex and suicidal ideation

Considering first the entire sample of athletes and non-athletes, a significantly higher percentage of female patients reported a positive ASQ compared to male patients (70.94% vs. 29.06% respectively, *p =* 0.0115; OR = 0.47, CI 95% [0.21, 0.99]). Unlike the findings from the overall population analysis, sex was not a significant predictor of suicidality within the athlete cohort (*p* = 0.0561). Similarly, amongst nonathletes, sex was not a significant predictor of suicidality (*p* = 0.0507).

### 3.5 Sport participation and suicidal ideation

When comparing ASQ responses between athletes and non-athletes, a significantly higher proportion of non-athletes screened positive for suicidal ideation (4.01%, *n* = 29) compared to athletes (1.05%, *n* = 60; *p* < 0.0001). No significant difference was found in suicidal ideation rates between athletes who participated in individual sports compared to those who participated in team sports (*p* = 0.5628). No other significant differences were found between groups amongst either demographic or sports variables, including the athlete type (multi- vs. single-sport athlete; Table 4).

**Table 4:** Clinical variables and sports characteristics in ASQ positive athletes.

Variable	*N* (%)	*p*-value
Visit provider		
Non-operative sports medicine physician	46 (76.67)	0.2102
Sports medicine surgeon	13 (21.67)	
Sports medicine neurologist	1 (1.67)	
Reason for visit—body part^**^		
Knee	29 (48.3)	N/R^****^
Shoulder/Collarbone	7 (11.7)	
Ankle	6 (10.0)	
Multiple Locations	6 (10.0)	
Hip	4 (6.7)	
Reason for visit—complaint		
Injury	26 (43.4)	0.7572
Pain	33 (55.0)	
Both injury and pain	1 (1.67)	
Primary diagnosis^**^		
Knee injury	19 (31.7)	N/R^****^
Knee pain	8 (13.3)	
Ankle injury	4 (6.67)	
Back/spine pain	5 (8.33)	
Shoulder injury	5 (8.33)	
Chronicity^***^		
Chronic	21 (40.4)	0.1453
Acute	31 (59.6)	
Competition level		
Recreational	12 (23.1)	0.0073^*^
School	23 (44.2)	
Club/Select/Travel	14 (26.9)	
Sport Participation type		
Individual sport	18 (30.0)	0.5628
Team sport	27 (45.0)	
Both individual and team sport	15 (25.0)	
Athlete type		
Multi-sport athlete	26 (43.3)	0.7962
Single-sport athlete	34 (56.7)	

* Indicates significance at α = 0.05.

** “Reason for Visit—Body Part” and “Primary Diagnosis” categories consist of the top 5 most reported body parts and diagnoses, respectively.

*** >90 days since injury is considered to be “chronic”.

**** N/R = no p-value was recorded for this variable.

**Figure 1:**
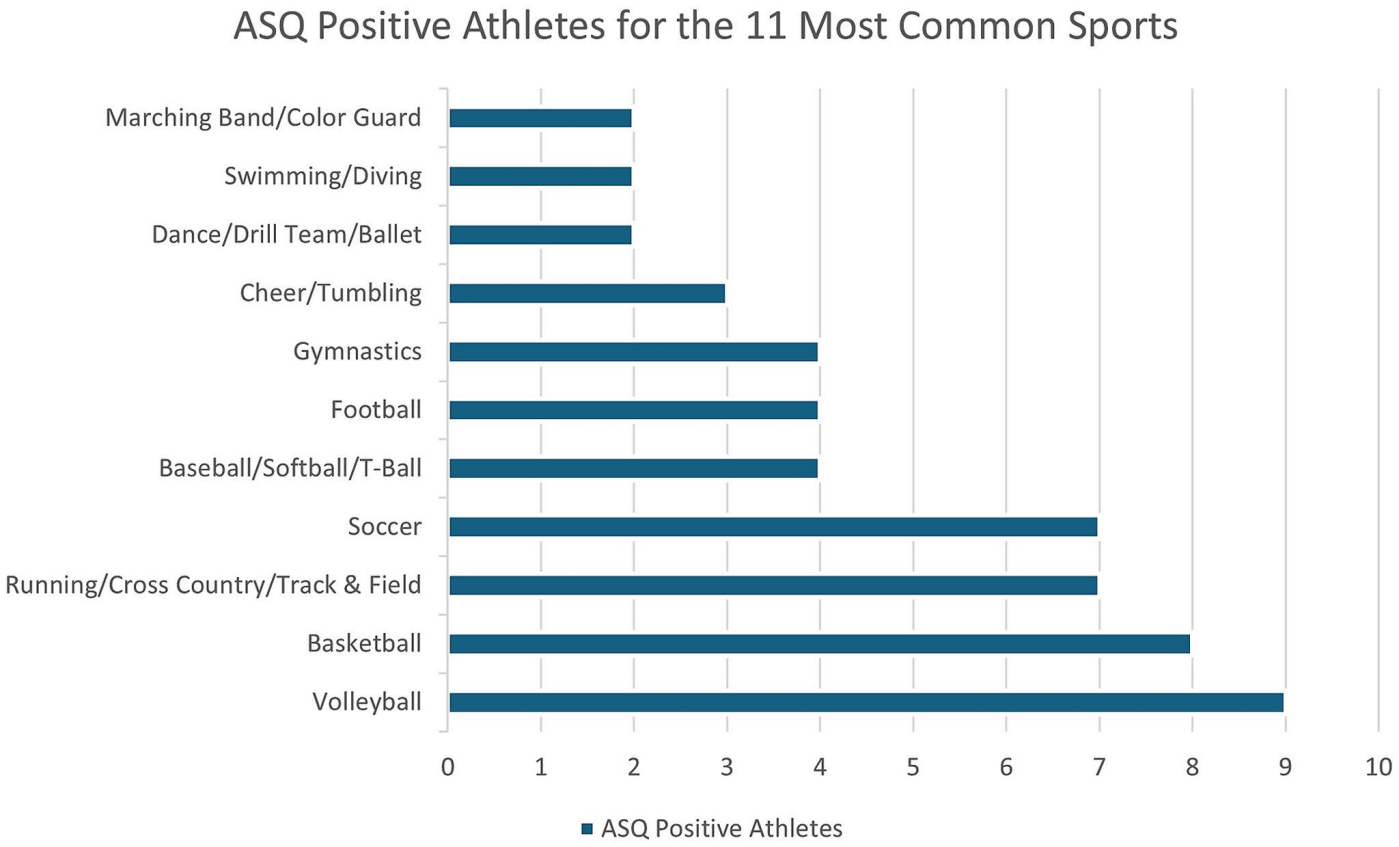
Ask suicide-screening questionnaire (ASQ) positive scoring athletes by primary sport for the 11 most common sports.

Among youth athletes with positive ASQ responses, volleyball and basketball were the most common primary sports, representing 15 and 13.3%, respectively (Figure 1). Marching Band/Color Guard had the highest proportion of ASQ positive responses (2 of 29 patients, 6.9%), followed by Running/Cross Country/Track and Field (7 of 301 patients, 2.3%), Golf (1 of 44 patients, 2.3%), and Volleyball (9 of 404 patients, 2.2%). Athlete competition level was significantly associated with ASQ outcomes (*p* = 0.0131). Post-hoc analyses comparing recreational and school athletes to club, select, and travel athletes revealed a significant difference in ASQ positive responses (*p* = 0.0029). Athletes participating in lower levels of competition (recreational or school) were more likely to screen ASQ positive compared to those participating in high levels of competition (club, select, travel; OR = 2.68, 95% CI [1.25, 6.40], *p* = 0.0162). The prevalence was highest in recreation (2.1%), followed by the school (1.4%) and club/select/travel level (0.6%).

## 4 Discussion

Outpatient medical clinics are increasingly becoming critical settings for regular screening for suicidality, as research shows that most individuals who die by suicide have seen a healthcare provider in the weeks or months prior (Ahmedani et al., 2014). Universal screening, which is a more comprehensive strategy than “targeted screening,” ensures that all youth, regardless of their presenting health issues, are assessed for suicide risk, reducing the chance that at-risk individuals may go unnoticed (Pediatrics, 2023). This study aimed to contribute to the ongoing discussion of mental health in pediatric patients diagnosed with sports medicine-related orthopedic conditions, with a focus on identifying the prevalence of suicidality. The primary goals were to investigate the prevalence of suicidality in this population and to discern if the prevalence varied based on specific diagnostic factors, demographic variables, or sport participation. The main findings indicate that sex assigned at birth and competition level were strong predictors of suicidality amongst pediatric patients seeking treatment in the sports medicine clinic.

The results of this study suggest that sex is a significant predictor of suicidality in the overall population; however, amongst athletes, sex is no longer a significant predictor of suicidality. Females were significantly more likely to screen positive for suicidal ideation than males, but in the subgroup of athletes, this difference disappeared, indicating that factors other than sex may be affecting mental health. Current literature supports these findings, that females are significantly more likely to report suicidal ideation than males (Miranda-Mendizabal et al., 2019; Zhang et al., 2019). Although research has shown that sports can serve as a protective factor against suicidal ideation among adolescents (Babiss and Gangwisch, 2009; Huo et al., 2024; Zuckerman et al., 2021), evidence on how gender differences affect the protective role of sports remains limited. Our findings suggest that athletic participation might play a role in mitigating some of the sex-related risks of suicidal ideation. These findings highlight the complexity of mental health in pediatric populations, showing that factors like sex and sport participation interact in ways that require a detailed and careful approach to care.

Competition level emerged as a significant predictor of suicidality, with lower rates observed among youth participating in club/select/travel levels compared to those engaged in school or recreational levels. These findings suggest that higher levels of competition in sports may act as a protective factor against suicidality. Specifically, a trend was observed among athletes: 2.1% of recreational athletes, 1.4% of school-level athletes, and only 0.6% of club/select/travel athletes screened positive on the ASQ, further indicating that the risk of suicidal ideation decreases as the level of competition increases.

However, there is conflicting evidence regarding these findings. Some studies highlight the negative effects of sports on young athletes, with higher competition levels being associated with increased stress and pressure, potentially contributing to anxiety and depression (Johnson and Waicus, 2015; Neal et al., 2015). For instance, in a study of elite Canadian swimmers, 68% of the surveyed athletes met the criteria for a major depressive episode, with females being particularly at risk (Yang et al., 2007). Moreover, the risk of injury in sports can have a serious negative impact on athletes’ identities, which tend to be more strongly tied to their athletic roles at higher competition levels (Choudhury et al., 2024). According to a study conducted by the NCAA, 33% of injured athletes reported high levels of depressive symptoms compared to 27% of non-injured athletes (Brewer and Petrie, 1995).

While competitive sports do present certain risks, many argue that the benefits often outweigh these concerns. Choudhury et al. (2024) found that athletes participating in higher competition levels, such as club or select teams, exhibited stronger overall athletic identity (the degree to which an individual identifies with the role of being an athlete), social identity (the athletic identity component focused on the personal connection an individual has to the athlete role), and negative affectivity (the athletic identity component focused on the emotional impact on an individual should an unwanted or negative sporting outcome occur) compared to those in lower competition levels, like recreational or school teams. The increased dedication and training demands at higher competition levels can lead to a more robust athletic identity, particularly in social and emotional contexts. This strengthened athletic identity does not necessarily diminish other aspects of self-identity. Athletes may continue to maintain a diversified self-identity, which can be crucial in protecting their sense of self during periods of injury or setback in sports.

Adolescents who perceive themselves as capable athletes may experience enhanced self-worth, serving as a protective factor against mental health concerns. Additionally, participation in team sports fosters strong relationships with teammates, providing a sense of belonging and reducing feelings of loneliness—both of which are commonly associated with mental health challenges. The sense of belonging, discipline, and support networks often found in more competitive sports environments may contribute to improved mental health outcomes. This underscores the potential benefits of encouraging youth participation in organized, competitive sports, particularly for those at risk for mental health issues.

The limitations of this study include the small sample size of those with a positive ASQ, which may have reduced the statistical power to detect significant differences or interactions between other variables. Additionally, the cohort’s generalizability is limited, as it is not fully representative of the broader orthopedic population or the general population; the study focused on patients presenting at a sports medicine clinic. While not all patients who present in the sports medicine clinic identify as athletes, it is expected that most are involved in some form of sport or athletic activity. Another limitation is the reliance on self-reported measures for suicidality and sports participation, which could introduce bias, as patients might underreport or overreport their symptoms or involvement due to social desirability or recall bias. A limitation of the self-reported measures was the inability to quantify athletes’ time spent in each sport when participating in multiple disciplines. Consequently, we analyzed data based on participation in individual, team, or both types of sports, which may lead to an inaccurate reflection of an athlete’s dominant athletic environment who is primarily engaged in one sport type but occasionally participates in the other. Future research could prioritize collecting data on time allocated across different sporting activities. Furthermore, since only the ASQ was required for inclusion in the study, incomplete or missing data from other forms or tests could affect the overall data completeness.

The findings from this study identify the importance of considering both psychological factors and sport participation in assessing suicidality among pediatric sports medicine patients. Specifically, the protective effect of higher competition levels in sports suggests that engagement in club/select/travel-level sports might play a role in reducing the risk of suicidal ideation. Future research should continue to explore the interplay between mental health, sport participation, and suicidality, particularly across different competition levels and in more diverse orthopedic populations. Such studies could inform targeted interventions and rehabilitation strategies aimed at improving both the psychological well-being and the athletic involvement of adolescents recovering from orthopedic injuries or ailments.

## Data Availability

The raw data supporting the conclusions of this article will be made available by the authors, without undue reservation.
